# Low-Dose Doxycycline Treatment Normalizes Levels of Some Salivary Metabolites Associated with Oral Microbiota in Patients with Primary Sjögren’s Syndrome

**DOI:** 10.3390/metabo11090595

**Published:** 2021-09-03

**Authors:** Maria Herrala, Soile Turunen, Kati Hanhineva, Marko Lehtonen, Jopi J. W. Mikkonen, Hubertus Seitsalo, Reijo Lappalainen, Leo Tjäderhane, Raija K. Niemelä, Tuula Salo, Sami Myllymaa, Arja M. Kullaa, Olli Kärkkäinen

**Affiliations:** 1 Research Group of Oral Health Sciences, Faculty of Medicine, Oulu University Hospital, University of Oulu, 90220 Oulu, Finland; arja.kullaa@uef.fi; 2 Institute of Dentistry, Faculty of Health Sciences, University of Eastern Finland, 70211 Kuopio, Finland; jopi.mikkonen@uef.fi; 3 Medical Research Center Oulu, Oulu University Hospital, University of Oulu, 90014 Oulu, Finland; leo.tjaderhane@helsinki.fi; 4 School of Pharmacy, University of Eastern Finland, 70210 Kuopio, Finland; soile.turunen@uef.fi (S.T.); marko.lehtonen@uef.fi (M.L.); olli.karkkainen@uef.fi (O.K.); 5 Institute of Public Health and Clinical Nutrition, University of Eastern Finland, 70211 Kuopio, Finland; kati.hanhineva@uef.fi; 6 Department of Life Technologies, Food Chemistry and Food Development Unit, University of Turku, 20014 Turku, Finland; 7 Department of Applied Physics, University of Eastern Finland, 70211 Kuopio, Finland; reijo.lappalainen@uef.fi (R.L.); sami.myllymaa@uef.fi (S.M.); 8 Private Dental Clinic Hammas&Hammas, 00130 Helsinki, Finland; hubertus.seitsalo@hammashammas.fi; 9 Department of Oral and Maxillofacial Diseases, Faculty of Medicine, University of Helsinki, 00014 Helsinki, Finland; tuula.salo@oulu.fi; 10 Department of Rheumatology, Oulu University Hospital, 90220 Oulu, Finland; Raija.Niemela@ppshp.fi; 11 Translational Immunology Research Program (TRIMM), University of Helsinki, 00014 Helsinki, Finland; 12 Department of Pathology, Faculty of Medicine, University of Helsinki, Helsinki University Hospital, 00014 Helsinki, Finland; 13 Cancer and Translational Medicine Research Unit, University of Oulu, 90014 Oulu, Finland; 14 Diagnostic Imaging Center, Kuopio University Hospital, 70029 Kuopio, Finland; 15 Educational Dental Clinic, Kuopio University Hospital, 90220 Kuopio, Finland

**Keywords:** saliva, Sjögren’s syndrome, metabolomics, hyposalivation, doxycycline

## Abstract

Saliva is a complex oral fluid, and plays a major role in oral health. Primary Sjögren’s syndrome (pSS), as an autoimmune disease that typically causes hyposalivation. In the present study, salivary metabolites were studied from stimulated saliva samples (*n* = 15) of female patients with pSS in a group treated with low-dose doxycycline (LDD), saliva samples (*n* = 10) of non-treated female patients with pSS, and saliva samples (*n* = 14) of healthy age-matched females as controls. Saliva samples were analyzed with liquid chromatography mass spectrometry (LC-MS) based on the non-targeted metabolomics method. The saliva metabolite profile differed between pSS patients and the healthy control (HC). In the pSS patients, the LDD treatment normalized saliva levels of several metabolites, including tyrosine glutamine dipeptide, phenylalanine isoleucine dipeptide, valine leucine dipeptide, phenylalanine, pantothenic acid (vitamin B5), urocanic acid, and salivary lipid cholesteryl palmitic acid (CE 16:0), to levels seen in the saliva samples of the HC. In conclusion, the data showed that pSS is associated with an altered saliva metabolite profile compared to the HC and that the LLD treatment normalized levels of several metabolites associated with dysbiosis of oral microbiota in pSS patients. The role of the saliva metabolome in pSS pathology needs to be further studied to clarify if saliva metabolite levels can be used to predict or monitor the progress and treatment of pSS.

## 1. Introduction

Saliva is an essential biofluid in the oral cavity, and its composition and volume are important factors in oral health. Saliva is secreted from three pairs of major salivary glands and several minor salivary glands, and the autonomic nervous system is involved in the control of salivary secretion [1] (pp. 27–29). Many factors, including systemic diseases, can affect salivary production. Sjögren’s syndrome (SS) is a disease that affects salivary glands, manifesting as hyposalivation and abnormal levels of salivary components [2]. Sjögren’s syndrome can be classified as primary (pSS) or secondary (sSS) forms. The focus of this article is pSS. SS has a wide range of clinical manifestations and symptoms, from affecting salivary or lacrimal glands to multi-organ symptoms and, potentially, a high risk of malignant lymphomas [3,4]. Patients with pSS usually have to wait a long time for a diagnosis, during which the disease progresses. Hauck et al. 2013 [5] noted a diagnosis delay of four years (median) between onset and diagnosis (range 0–28 years) in a Canadian population. There is an urgent need to study and develop new tools for the diagnosis and monitoring of pSS.

The metabolic profile of saliva can provide an early diagnosis of pSS and monitoring of its progress [6]. SS is associated with alterations in the metabolite profile of saliva; for example, elevated levels of choline, taurine, and alanine have been reported [6,7]. Recent developments in instrumentation have led to new spectrometric platforms for metabolomics, which employ state-of-the-art analytical techniques, such as different mass spectrometry methods in conjunction with either high-performance liquid chromatography (HPLC-MS) or two-dimensional gas chromatography (2DGC-MS) and nuclear magnetic resonance (NMR) spectroscopy [8]. In our previous study, 24 salivary metabolites were identified using ^1^H NMR spectroscopy [9].

Gardner et al. 2020 [10] listed numerous diseases that have been studied using metabolomic techniques, for which potential salivary biomarkers have been found. These include oral cancer, oral leukoplakia, breast cancer, prostate cancer, periodontal disease (common, aggressive, and chronic), dental caries, pSS, dementia, Alzheimer’s disease, mild cognitive impairment, Parkinson’s disease, celiac disease, sarcoidosis, recurrent aphthous ulceration, untreated and treated HIV, hepatitis B, medication-related osteonecrosis of the jaw, parotid gland tumor, adult and pediatric obesity, and external apical root resorption in orthodontic therapy. There is new knowledge indicating that dysbiotic oral microbiota can invade the ductal cells, providing new insights into the etiopathogenesis of pSS [11].

Matrix metalloproteinases (MMPs) have been studied as a potential target of interest in the treatment plan of pSS. Low-dose doxycycline (LDD) has been suggested to help pSS patients’ symptoms by decreasing MMP activity. LDD also has an antimicrobial effect [12]. In Seitsalo et al. [12], data suggested that LDD medication did not relieve pSS patients’ symptoms.

The present study aimed to measure changes in the saliva metabolite profile associated with pSS and to investigate if LDD treatment can normalize some of these changes. We used non-targeted LC-MS-based metabolomics methods to measure saliva metabolite profiles from samples collected from healthy controls (HC) and pSS patients with or without LDD treatment.

## 2. Results

**Figure 1:**
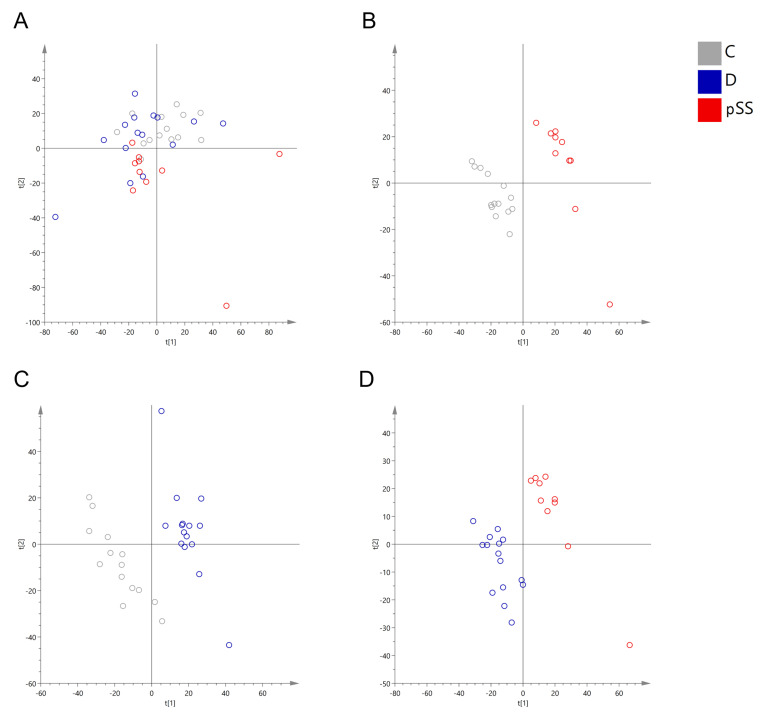
Results of principal component analysis of the metabolomics data. In the principal component analysis (PCA), a separation of the groups can be seen, in which the metabolite profile of the saliva samples from the Sjögren’s syndrome patients before drug treatment (pSS) are separated from the metabolite profile of saliva samples from the HC (C), whereas Sjögren’s syndrome patients with LDD treatment (D) are mixed with the first two groups (**A**). Partial least sum of squares (PLS-DA) models between the controls and the Sjögren’s syndrome patients without drug treatment ((**B**): Three components, R2Y (cumulative) = 0.99, Q2 (cumulative) = 0.77), between the controls and Sjögren’s syndrome patients with low-dose doxycycline treatment ((**C**): Four components, R2Y (cum) = 0.99, Q2 (cum) = 0.67), and Sjögren’s syndrome patients with and without drug treatment ((**D**): Two components, R2Y (cum) = 0.94, Q2 (cum) = 0.39).

The salivary flow rate was 0.14 ± 0.06 mL/min (mean ± SD) before medication and 0.15 ± 0.07 mL/min after one week of medication. No statistically significant difference was observed between salivary flow rates. All identified metabolites with *p*-values below 0.05 are reported in Table 1. The multivariate analysis results are shown in Figure 1. The PCA showed the separation of the untreated pSS patients’ saliva metabolite profile from the saliva metabolite profile of the HC. pSS patients with LDD treatment were mixed with the first two groups in the PCA, indicating that the doxycycline treatment shifted the saliva metabolite profile closer to that seen in the HC compared to the untreated pSS patients.

Good predictability is seen in the PLS-DA model between the saliva metabolite profiles of the HC and the untreated pSS patients (PLS-DA model with three components, Q2 (cumulative) = 0.77), indicating a clear separation of the saliva metabolite profiles, in line with the PCA results. In contrast, the predictability was decreased in the PLS-DA models between the LDD-treated pSS patients’ salivary metabolite profiles compared to the HC (Q2 (cum) = 0.67) and the untreated pSS patients (Q2 (cum) = 0.39). However, the results also show that, even after the LDD treatment, the saliva metabolite profile of pSS patients is different from that of HC.

Identified metabolites that were significantly different between the saliva samples of patients with pSS and HC are reported in Table 1. The results of the univariate analyses were mostly in line with the results of the multivariate analysis. The exceptions were lysophosphatidylethanolamide 18:0 (LPE 18:0) and lysophosphatidylcholine 18:0 (LPC 18:0), which were not observed in the samples from HC, but were seen in most of the samples from patients with pSS. VIP values from PLS-DA, and *p*-values and Cohen’s d effect sizes, are reported in Table 1. In the univariate analysis, there were 912, 767, and 223 molecular features with *p*-values below 0.05 when comparing untreated pSS patients to controls, LDD-treated pSS patients to controls, and untreated pSS patients to treated pSS patients, respectively. However, we were not able to identify most of these molecular features, which is typical for a non-targeted metabolomics study. In the pathway analysis, four pathways had *p*-values below 0.05: aminoacyl-tRNA biosynthesis (*p* = 0.0013); valine, leucine, and isoleucine biosynthesis (*p* = 0.0082); nicotinate and nicotinamide metabolism (*p* = 0.0286); and histidine metabolism (*p* = 0.0323) when the pSS group was compared to the controls. However, none of these results survived correction for multiple testing (Supplementary Table S1).

**Figure 2:**
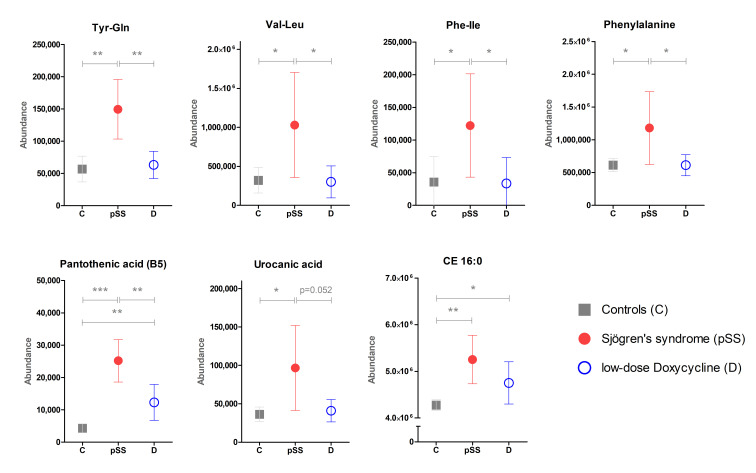
LDD treatment normalized levels of metabolites in the saliva samples of patients with Sjögren’s syndrome. Several metabolites were altered in the saliva samples of patients with pSS when compared to the saliva samples from HC. LDD treatment normalized levels of some, but not all, of these metabolites closer to levels seen in HC. Mean ion abundance with 95% confidence intervals is shown. Legend: CE 16:0, cholesteryl palmitic acid; Phe-Ile, phenylalanine isoleucine dipeptide; Tyr-Gln, Tyrosine glutamine dipeptide; Val-Leu, Valine leucine dipeptide; *, *p* < 0.05; **, *p* < 0.01; ***, *p* < 0.001.

In the pSS patients, the LDD treatment normalized saliva levels of some metabolites, namely, tyrosine glutamine dipeptide, phenylalanine isoleucine dipeptide, valine leucine dipeptide, phenylalanine, pantothenic acid (vitamin B5), urocanic acid, and cholesteryl palmitic acid (CE 16:0), to levels seen in the saliva samples of the HC (Figure 2). Here, the results of multivariate and univariate analyses are also in line.

## 3. Discussion

In the present study, we observed that the saliva metabolite profile was different between pSS patients and HC. Moreover, we found out that the LDD treatment reversed some, but not all, of these changes.

We observed high levels of dipeptides, namely, tyrosine glutamine dipeptide, phenylalanine isoleucine dipeptide, and valine leucine dipeptide, and phenylalanine, in the saliva samples of patients with pSS. Previous reports have shown that pSS patients have dysbiosis in oral microbiota in bacterial species (e.g., Prevotella and Porphyromonas species) that can degrade proteins into smaller peptides and further into amino acids, such as phenylalanine [11,13,14,15,16,17]. Moreover, high levels of dipeptides and phenylalanine in saliva have been associated with dysbiosis of oral microbiota in other oral pathologies, such as periodontitis [15,18]. Therefore, high levels of dipeptides and phenylalanine in the saliva of pSS patients are likely to be associated with dysbiosis in the oral microbiota composition.

Moreover, high pantothenic acid (vitamin B5) levels were observed in saliva samples of pSS patients. Oral microbiota can synthesize pantothenic acid, which is needed to form coenzyme-A (CoA), an essential cofactor of cellular metabolism [19,20,21]. Pantothenic acid has been associated with the release of cytokines, and appears to have antibacterial properties towards some bacteria, such as mycobacteria [22,23]. Urocanic acid can also be degraded by bacteria [24]. Therefore, high levels of pantothenic acid and urocanic acid in the saliva of pSS patients may also be associated with dysbiosis in the oral microbiota composition.

Furthermore, LDD treatment normalized levels of several metabolites associated with dysbiosis of the oral microbiota (Figure 2). In the present study, we analyzed masticatory stimulated saliva, which is verified as an adequate alternative to unstimulated saliva for microbiome-related studies [25]. LDD treatment did not show a statistically significant difference for salivary flow. However, it is unclear how or if dysbiosis is connected to pSS. Therefore, it appears likely that LDD treatment normalized the dysbiosis of oral microbiota seen in pSS patients, a hypothesis that needs further investigations.

Moreover, cholesteryl palmitic acid (CE 16:0) is an ester of cholesterol found in saliva, in addition to cell membranes and blood [26]. Salivary lipids are important for the flexibility, fluidity, and permeability of oral cellular membranes, and levels of salivary lipids, including cholesteryl esters, are altered in SS patients [27,28]. Therefore, LDD appears to normalize the levels of some salivary lipids, i.e., CE 16:0 in this study, but not all in pSS patients.

The pathway analysis indicated that four pathways, namely aminoacyl-tRNA biosynthesis; valine, leucine, and isoleucine biosynthesis; nicotinate and nicotinamide metabolism; and histidine metabolism, were altered in the saliva samples from the pSS group when compared to the controls, indicating alterations in the amino acid metabolism. However, these results should be considered preliminary and be verified with a larger cohort of patients.

It should be noted that the LDD treatment appears to only normalize the saliva metabolite profile in some patients with pSS (Figure 1). Unfortunately, the present study had limited statistical power, due to a relatively small sample size. Therefore, we were not able to conduct subgroup analyses to compare those who responded well to LDD treatment to those who did not. With a larger sample size, this kind of pharmacometabolomics analysis may reveal new predictive biomarkers to recognize, before treatment, those individuals who are most likely to benefit from the LDD treatment. In Seitsalo et al. [12], LDD did not affect pSS patients’ clinical symptoms.

In conclusion, we observed that pSS is associated with an altered saliva metabolite profile when compared to HC. Furthermore, we showed that the LDD treatment normalized levels of several metabolites associated with dysbiosis of oral microbiota in patients with pSS. Further study is needed to better understand the role of the saliva metabolome in pSS pathology and to investigate if saliva metabolite levels can be used to predict patients who are likely to benefit from doxycycline treatment.

## 4. Materials and Methods

### 4.1. Ethical Statement

This study was designed within the recommendations of the Declaration of Helsinki and the Oulu University Hospital Ethical Committee gave a favorable opinion regarding all plans of the study (EETTMK: 116/2000 and 36/2012). For the mass spectroscopy component of the study, ethical permission for the research was granted by The Hospital District of Northern Savo, Kuopio, Finland (745/2018; (82/2014). As stated in the Declaration of Helsinki, all participants give their written consent to participate in this study.

### 4.2. Participants

The pSS group consisted of 15 female patients who were aged 28–68 years (mean age 48.6 years). Diagnostic criteria for Sjogren’s syndrome were based on those of the European Community guidelines [29,30]. Smoking habit, and oral or systemic diseases other than pSS, were exclusion criteria; more detailed information is provided in Niemelä et al., (2004).The control group consisted of 14 healthy females, aged between 28 and 68 years (mean age 49.8 years). The control subjects did not have chronic diseases, were non-smoking, and were not receiving treatment to affect the results.

### 4.3. Collection of Salivary Samples

Both stimulated and unstimulated saliva samples from the pSS patients were collected as described in Seitsalo et al. [12]. When saliva samples were collected, no periodontal diseases or carious lesions were present. Because patients typically suffer from hyposalivation, some samples were not sufficient for analysis with LC-MS or were poor quality. Saliva flow rates (mL/min) were calculated immediately after collection, as described previously (Herrala et al., (2021)). The Wilcoxon signed ranks test was used to analyze the salivary flow rate between the pre-medication and one week medication samples. Therefore, from a total of 25 stimulated saliva samples of pSS patients were analyzed: 10 before LDD treatment and 15 after treatment with LDD (Periostat ^R^, 20 mg doxycycline), which was given twice per day for one week. The control group saliva samples (*n* = 14) were collected only once at the medical campus of the University of Oulu, Finland.

**Figure 3:**
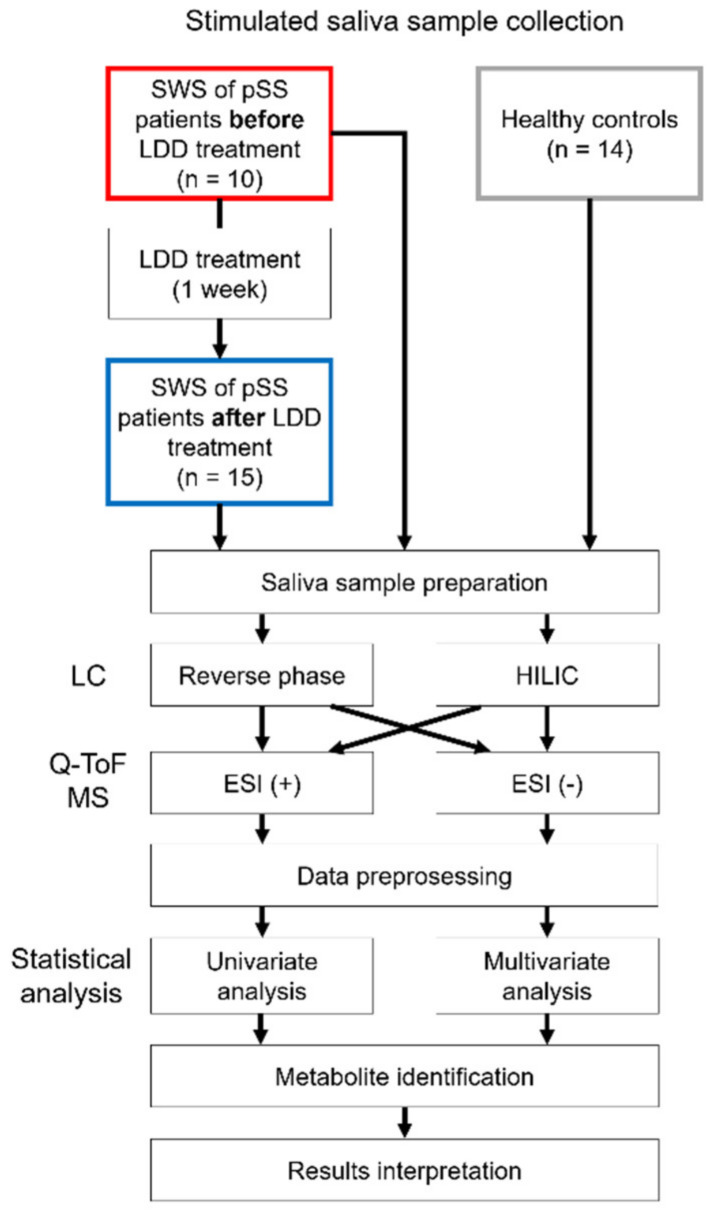
Workflow of the non-targeted metabolomics analysis. Stimulated whole saliva (SWS) samples were collected from patients with primary Sjögren’s syndrome (pSS) before and after low-dose doxycycline (LDD) treatment (20 mg twice per day for one week) and from healthy controls. Saliva samples with low quantity or low quality were removed from the analysis. Details of the non-targeted metabolomics analysis of saliva samples has been previously described [32]. Briefly, after sample preparation, the samples were analyzed with four different analytical methods: using both reverse phase and hydrophilic interaction (HILIC) liquid chromatography (LC) separation, followed with both positive and negative electrospray ionization (ESI). After data preprocessing, we used both multivariate and univariate statistical methods to identify molecular features of interest, from which identification of metabolites was undertaken using both in-house and publicly available databases.

The saliva sample collection followed the protocols proposed in the study of Navazesh 1993 [31]. All the saliva samples were collected considering the circadian rhythm (between 10 am and 12 am). Eating and drinking were not allowed a minimum of one hour before the saliva sample collection. After collection, the saliva samples were immediately centrifuged and the supernatants were stored and transported as described in [6,9]. We demonstrate this study design in Figure 3.

### 4.4. Metabolomics Analysis

The metabolomics analysis pipeline and the saliva sample metabolomics analysis have been previously described in detail [32,33]. Briefly, the saliva samples were thawed on ice. Saliva samples were precipitated and extracted in the ratio of 200 µL of saliva and 400 µL of acetonitrile. All samples were centrifuged (10,600× *g*, 5 min, +4 °C), and the supernatants were filtered through 0.2 µm Acrodisc^®^ Syringe Filters with a PTFE membrane (PALL Corporation, Ann Arbor, MI, USA) prior to the LC-MS analyses. The quality control (QC) sample contained 30 µL aliquots from all experimental samples mixed in one tube.

HPLC-grade acetonitrile (VWR Chemicals, Fontenay-sous-Bois, France) was used for sample preparation. LC-MS grade methanol (Riedel-de Haën™, Honeywell, Seelze, Germany), HPLC-grade acetonitrile (VWR Chemicals, Fontenay-sous-Bois, France), LC-MS grade formic acid (Fluka™, Honeywell, Seelze, Germany), ammonium formate (Fluka™, Honeywell, Seelze, Germany), and class 1 ultra-pure water (ELGA Purelab ultra Analytical, High Wycombe, UK) were used for mobile phase eluents in reverse phase (RP) and hydrophilic interaction (HILIC) liquid chromatography separation.

The samples were analyzed by a 1290 LC system coupled to a 6540 UHD accurate-mass Q-ToF spectrometer (Agilent Technologies, Waldbronn, Karlsruhe, Germany) using electrospray ionization (ESI, Jet Stream) in both positive and negative polarity, and using both RP and HILIC.

For the quality assurance of the chromatographic and mass spectrometry runs, QC samples were injected at the beginning of the analysis and after every 9 samples. The QC samples were used for the automatic data-dependent MS/MS analyses. The data acquisition was accomplished with MassHunter Acquisition B.05.01 software (Agilent Technologies).

The LC-MS raw data from four different analytical modes (RP+, RP−, HILIC+, HILIC−) was exported to MassHunter Qualitative Analysis B.07.00 (Agilent Technologies, Santa Clara, CA, USA) for feature extraction and peak picking, combined with chromatographic alignment across all data files per mode. To remove the redundant and non-specific information considered to be background noise, peaks with ion abundance less than 10,000 were excluded from further analysis. The feature files were imported as compound exchange format (.cef) files into Agilent Mass Profiler Professional software (MPP version 13.1.1, Agilent Technologies) for compound alignment to yield a peak list.

Multivariate analyses, principal component analysis (PCA), and partial least sum of squares discriminant analysis (PLS-DA) were performed to mean centered and autoscaled data using SIMCA (version 15, Umetrics, Umea, Sweden). For univariate analysis, we used Cohen’s d to calculate effect sizes and Welch’s t-test to calculate *p*-values from non-scaled ion abundance data. Because of the correlated nature of metabolomics data, we adjusted the α level by the number of latent components needed to explain 95% of the variance in the metabolomics data in the PCA to account for multiple testing. Here, 32 latent components were needed to explain 95% of the data and the new α was set to 0.002.

The metabolite identification was performed using open-source software, MS-DIAL (RIKEN PRIMe). Collected MS/MS data was converted to .abf files using the Analysis Base File Converter program (Reifycs Inc., Tokyo, Japan) and converted files were imported into MS-DIAL (versions 2.66 to 3.90). Public databases, Metlin and MassBank of North America (MoNA), and an in-house standard library were used. The guidelines from Sumner et al. [34] were used for ranking metabolite identifications as follows: Compounds in identification level 1 were verified by comparing exact mass, retention time, and MS/MS fragmentation spectra with the in-house standard library. Compounds in level 2 were matched with exact mass and MSMS spectra from the public databases mentioned above. We used MetaboAnalyst (version 5.0, [35]) to undertake a pathway analysis of identified metabolites with a *p*-value below 0.05. We used KEGG pathways for humans as a reference metabolic pathway.

## Figures and Tables

**Table 1:** Identified salivary metabolites associated with Sjögren’s syndrome.

Metabolite	ID	Healthy Controls, HC (C)		Sjögren’s Syndrome (pSS) without LDD		Sjögren’s Syndrome (pSS) with LDD (D)		pSS vs. C			D vs. C			D vs. pSS		
		Mean	SD	Mean	SD	Mean	SD	VIP	*p*	d	VIP	*p*	d	VIP	*p*	d
Amino acids, peptides, and analogues																
4-Guanidinobutyric acid	2	16,864	4783	31,881	18,325	28,463	10,929	1.38	0.031	1.3	1.83	0.004	1.5	0.39	0.612	−0.2
Glutamic acid	1	122,045	67,786	250,491	87,720	225,246	124,395	1.91	0.001	1.7	1.48	0.010	1.1	0.72	0.558	−0.2
Glycine betaine	1	1241,617	504,031	1,999,692	964,523	1,849,530	974,498	1.43	0.041	1.0	1.31	0.045	0.8	0.33	0.708	−0.2
Isoleucine	2	48,927	30,704	166,871	135,723	99,906	78,292	1.54	0.023	1.4	1.28	0.037	0.9	1.00	0.184	−0.6
Leucine	2	112,620	46,369	448,733	419,771	190,974	115,700	1.56	0.032	1.4	1.37	0.026	1.0	1.54	0.088	−1.0
Phenylalanine	1	614,751	174,399	1,180,634	775,776	612,765	291,252	1.41	0.048	1.2	0.53	0.982	0.0	1.58	0.050	−1.1
Tryptophan	1	33,773	17,245	97,779	66,429	56,455	30,196	1.63	0.014	1.5	1.32	0.021	1.0	1.32	0.091	−0.9
Arg-Ser	2	653,047	308,815	285,658	248,390	447,258	269,746	1.56	0.004	−1.3	1.10	0.072	−0.7	1.49	0.145	0.6
Phe-Ile	2	35,793	41,934	122,220	85,371	33,595	37,875	1.55	0.040	1.4	0.39	0.923	−0.1	1.81	0.037	−1.4
Tyr-Gln	2	56,858	34,559	149,463	64,758	63,153	33,358	1.96	0.001	1.9	0.48	0.641	0.2	2.23	0.002	−1.8
Val-Leu	2	320,566	283,561	1,029,040	940,151	300,576	370,401	1.42	0.043	1.2	0.47	0.871	−0.1	1.61	0.040	−1.1
Lipids and carnitines																
FA 16:0	2	19,113,838	2,988,053	23,755,538	5,398,164	21,707,731	6,550,119	1.40	0.029	1.1	1.01	0.181	0.5	0.58	0.403	−0.3
FA 16:1	2	291,295	177,097	601,015	353,046	406,038	534,487	1.46	0.025	1.2	0.64	0.442	0.3	0.91	0.283	−0.4
Azelaic acid	1	46,004	5591	61,345	16,287	54,030	11,500	1.67	0.161	1.4	1.27	0.025	0.9	0.95	0.238	−0.5
Leucic acid	2	61,064	25,243	42,491	15,691	45,390	20,708	1.25	0.037	−0.9	1.00	0.089	−0.7	1.23	0.706	0.2
CE 16:0	1	4,277,005	204,081	5,253,366	726,120	4,751,349	817,147	2.03	0.002	2.1	1.23	0.045	0.9	1.02	0.122	−0.7
Cholesterol	1	42,086	23,976	107,189	65,001	111,790	89,827	1.66	0.118	1.5	1.50	0.010	1.2	0.45	0.883	0.1
Propionylcarnitine	1	91,310	57,056	165,515	81,484	202,565	108,289	1.47	0.025	1.1	1.73	0.002	1.3	0.62	0.340	0.4
Isobutyryl carnitine	2	50,626	23,350	95,837	32,915	123,268	75,870	1.85	0.002	1.6	1.80	0.004	1.5	0.77	0.244	0.5
LPC 18:0	2	0	0	24,441	13,129	71,932	99,287	0.01	<0.001		0.68	<0.001		0.84	0.170	0.8
LPE 18:0	2	0	0	16,498	8405	17,072	8133	0.34	<0.001		0.25	<0.001		0.35	0.941	0.1
Oleamide	2	9,389,628	1,079,571	11,488,697	1,386,541	10,258,327	2,115,434	1.85	<0.001	1.7	0.96	0.174	0.5	1.03	0.092	−0.7
Linoleamide	2	30,287,081	4,290,249	37,683,667	8,799,870	35,644,986	12,192,656	1.41	0.030	1.1	1.04	0.140	0.7	0.44	0.639	−0.2
Palmitoleamide	2	12,508,377	1,779,147	15,694,049	3,897,803	15,115,575	6,193,420	1.41	0.033	1.1	1.04	0.137	0.7	0.20	0.777	−0.1
Other																
Choline	2	5,453,217	2,359,520	9,987,031	4,308,942	9,524,364	4,603,084	1.59	0.010	1.4	1.60	0.009	1.2	0.31	0.804	−0.1
Pantothenic acid (B5)	1	4289	2431	25,186	9179	12,290	10,088	2.46	<0.001	3.6	1.53	0.009	1.3	1.81	0.003	−1.3
MEHP	1	101,276	15,673	58,944	15,552	88,751	27,838	2.35	<0.001	−2.7	0.87	0.146	−0.6	1.77	0.002	1.4
Xanthine	1	173,813	123,732	548,441	260,922	601,704	302,916	2.05	0.001	1.9	2.15	<0.001	2.0	0.41	0.644	0.2
Urocanic acid	1	36,386	16,273	96,702	77,360	40,992	25,246	1.47	0.037	1.3	0.41	0.572	0.2	1.55	0.052	−1.1
1-Methylnicotinamide	2	11,940	1594	28,433	16,638	33,923	33,704	1.68	0.039	1.8	1.27	0.069	1.2	0.42	0.664	0.2
Biliverdin IX	1	162,101	31,725	137,588	11,938	140,733	11,422	1.28	0.017	−1.1	1.33	0.030	−1.0	0.46	0.519	0.3
N6-methyl-adenine	2	55,816	27,354	119,280	86,943	102,200	74,734	1.35	0.049	1.1	1.35	0.037	0.9	0.39	0.618	−0.2
Nicotinic acid	1	43,642	31,275	107,135	80,169	87,564	60,411	1.37	0.049	1.1	1.34	0.029	1.0	0.71	0.541	−0.3
Diethanolamine	1	147,588	168,396	695,458	459,234	219,384	495,663	1.89	0.004	1.7	0.56	0.628	0.2	1.56	0.027	−1.0
Stearamide	2	10,065,816	1,374,684	12,518,622	3,182,570	11,313,506	3,775,837	1.35	0.042	1.1	0.94	0.247	0.5	0.56	0.399	−0.3
Dibutyladipate	2	73,783	33,110	43,796	6608	86,324	85,921	1.45	0.005	−1.5	0.54	0.606	0.2	1.10	0.077	0.9

Legend: Bolded text, *p*-values below multiple comparison corrected α (*p* < 0.002); Arg-Ser, Arginine serine dipeptide; CE 16:0, cholesteryl palmitic acid; d, Cohen’s d effect size; FA, fatty acid; ID, level of identification (1 = verified by comparing exact mass, retention time, and MS/MS fragmentation spectra with in-house standard library, 2 = matched with exact mass and MSMS spectra from public databases); LPC, lysophosphatidylcholine; LPE, lysophosphatidylethanolamine; MEPH, Monoethylhexyl phthalic acid; Phe-Ile, phenylalanine isoleucine dipeptide; SD, standard deviation; Tyr-Gln, Tyrosine glutamine dipeptide; Val-Leu, Valine leucine dipeptide; VIP, variable importance to projection (from PLS-DA); *p*, *p*-value from Welch’s *t*-test; Mean ion abundance is shown; Identified metabolites with *p* < 0.05 in comparison between patients with pSS and HC are shown.

## Data Availability

The data presented in this study are available in [Supplementary Table S1].

## Supplementary Materials

The following are available online at https://www.mdpi.com/article/10.3390/metabo11090595/s1, Table S1: Results of the pathway analysis.
